# Can extended upper pole ureterectomy prevent ureteral stump syndrome after proximal approach for duplex kidneys?

**DOI:** 10.1590/S1677-5538.IBJU.2020.0686

**Published:** 2021-02-28

**Authors:** Bruno Nicolino Cezarino, Roberto Iglesias Lopes, Ricardo Haidar Berjeaut, Francisco Tibor Dénes

**Affiliations:** 1 Universidade de São Paulo Faculdade de Medicina Hospital das Clínicas SP Brasil Unidade de Urologia Pediátrica, Divisão de Urologia, Hospital das Clínicas, Faculdade de Medicina da Universidade de São Paulo - FMUSP, SP, Brasil

**Keywords:** Cakut [Supplementary Concept], Ureter, Urinary Tract Infections

## Abstract

**Introduction:**

Symptomatic duplex kidneys usually present with recurrent urinary tract infection due to ureteral obstruction (megaureter, ureterocele or ectopic ureter) and/or vesicoureteral reflux. Upper-pole nephrectomy is a widely accepted procedure to correct symptomatic duplex systems with poor functioning moieties, also known as upper or proximal approach. The distal ureteral stump syndrome (DUSS) can be a late complication of this approach. There is no consensus upon the length of ureteral dissection and the better approach to symptomatic disease in duplex systems, so we aim to identify if extended ureteral dissection can prevent DUSS in top-down approach.

**Materials and Methods:**

Forty-four consecutive patients with symptomatic duplex system were retrospectively classified into two groups: those with limited ureteral excision after heminephrectomy (HN) (group-1) and those with extended ureterectomy after HN (group-2). Patients were followed-up for at least 36 months regarding outcomes of distal ureteral stump.

**Results:**

Overall complication was 20%. A total of 8 patients required unplanned further surgery in Group-1 (30%) whereas only 1 patient required unplanned surgery in group 2 (6%) (p=0.07). Subgroup analysis showed that Group-1 presented more DUSS requiring surgery during follow-up than group-2 (p=0.04). Factors possibly affecting complications incidence (such as ureterocele or ectopic ureter) did not differ between groups (p=0.72 and p=0.78).

**Conclusion:**

Upper pole nephrectomy should be performed with extended distal ureteral dissection to prevent ureteral stump complications.

## INTRODUCTION

Duplex kidneys are the most common congenital anomalies of the upper urinary tract, with an estimated incidence of 0.8% (1). Symptomatic duplex kidneys usually present with recurrent urinary tract infection due to ureteral obstruction of the upper pole, megaureter, ureterocele or ectopic ureter, and/or vesicoureteral reflux to the lower pole, eventually compromising affected moiety function (2, 3).

Upper-pole nephrectomy is a widely performed procedure to correct symptomatic duplex systems with poor functioning upper moieties. Alternatively, this condition can be managed with uretero-pyelic or uretero-ureteral anastomosis of the upper to the lower moiety, even in cases of low-function. Nowadays both procedures, also known as upper or proximal approaches, are usually performed by laparoscopic technique, with simple and straightforward techniques.

Another option is the lower or distal approach, which address directly the uretero-vesical junction (UVJ), whose main advantage is the more comprehensive correction of the anatomical abnormalities of both ureters, albeit with a significantly more invasive reconstructive surgery, which is also prone to more complications (4).

The distal ureteral stump syndrome (DUSS) can be a late manifestation of proximal approach and it is defined as any complication related to the upper pole ureteral stump: stump empyema or reflux, symptomatic febrile urinary tract infection (UTI), persistent bacteriuria or urethral discharge, hematuria or low quadrant pain, which frequently requires intervention (5, 6).

There is no consensus regarding the best choice between the proximal and distal approaches in the treatment of symptomatic disease in duplex systems, as well as the adequate length of ureteral dissection in the later technique (7, 8).

This study aims to identify if extended ureteral dissection can prevent DUSS in the upper or proximal approach.

## MATERIALS AND METHODS

An ethics committee approved (research protocol 1.895748) the study and a prospective database was maintained for consecutive patients undergoing surgery for duplex kidneys in our tertiary pediatric urology center between 2005 and 2017 and a retrospective analysis was performed. Upper-pole nephrectomy has been the standard of care in our institution for symptomatic non-functioning upper pole moieties in duplex kidneys. All patients underwent ultrasonography (US) and DMSA nuclear scan or computer tomography scan that confirmed the significantly impaired anatomy and function of the upper moiety, as well as a voiding urethrocystogram (VCUG) prior to surgery. Indications for surgery included urinary tract infection associated to vesical ureteral junction obstruction, urethral obstruction due to ureterocele and/or urinary incontinence. Endoscopic incision was not performed in patients with asymptomatic ureteroceles and was only used to decompress acutely infected ureteroceles.

Patients were classified into two groups: Group-1 underwent upper pole heminephroureterectomy (HN) with limited or no distal ureterectomy and Group-2 heminephroureterectomy (HN) with aggressive extended ureterectomy, well below iliac vessels. The ureteral stump was ligated or left open according to initial pathology of the affected moiety. The surgical access (laparoscopic vs. open) was chosen according to surgeon's preference and expertise, but favored extended ureterectomy after 2008, after reviewing our initial results that showed a DUSS rate of 15.7% (9).

Mean age at operation, baseline pathology, long-term complications related to ureteral stump and requirement for further surgical intervention were recorded for all cases. All patients were followed with US at 6 weeks and 6 months post-operatively, then on a yearly basis, with a minimum follow-up of 36 months. Only symptomatic patients underwent a postoperative repeat VCUG. Statistical analysis consisted of chi-square test and Mann-Whitney, with p value <0.05 being statistically significant.

### Operative technique details

**Figure 1:**
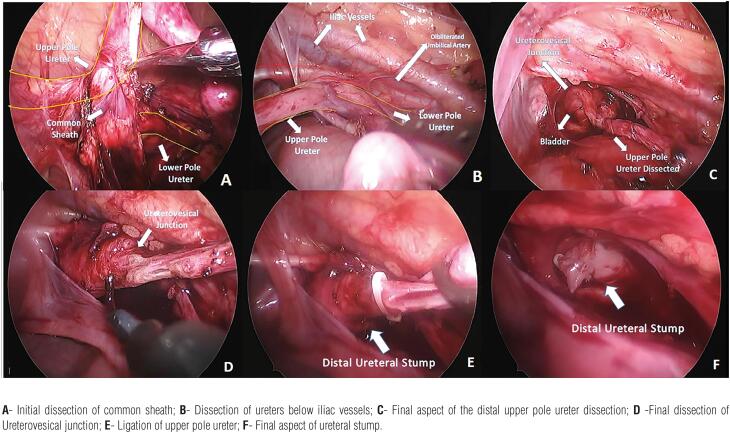
Laparoscopic dissection of distal ureter in a 4 years-old girl with duplex kidney with non-functioning reflux upper pole.

Briefly, upper-pole nephrectomy involves proximal ureteral dissection of affected moiety, ligation of polar vessels, division of two moieties and resection of the ischemic upper pole with electric or harmonic scalpel. Care is taken to remove all non-viable renal and collector tissue to prevent post-operative retention cysts. The extended distal ureterectomy, performed in Group-2, can be challenging due to the common sheath of the duplicated ureters (10). Laparoscopic approach precludes the need of another open incision to perform distal dissection (Figure-1). The careful dissection is extended close to the UVJ, avoiding lesion to the functional lower pole ureter (Figure-1). As mentioned above, ligation of the distal stump in both groups is based on baseline pathology, being obligatory in cases of VUR and optional for obstructive cases (Figure-1).

## RESULTS

A total of 44 upper pole nephrectomies were performed: 28 in Group-1 and 16 in group-2. Sex, diagnosis, indication for initial surgery and age at surgery were similar between groups. Two patients in group-1 and one patient in group-2 underwent ureterocele puncture before surgery and presented with secondary vesical ureteral reflux to upper pole. Median follow-up was significantly shorter among patient submitted to extended ureterectomy (42 months, with range of 36 - 108) compared to limited dissection (84 months, with a range of 72-184) (Table-1).

**Table 1:** Demographics, follow-up, unplanned surgery diagnosis and need of further surgery.

Group		1- Limited ureteral extension	2- Extended Ureteral Dissection	p value
**Sex**				
	F / M	26(92%) / 2(8%)	11(68%) / 4(32%)	p=0.08
**Diagnosis**				
	Upper pole ureterocele	14 (50%)	9 (60%)	0.72
	Secondary vur after puncture	2(7%)	1(6%)	0.64
	Primary UVJ obstruction	6(21%)	2(12%)	0.42
	Ectopic ureter	6(21%)	4(24%)	0.78
**Indication for initial surgery**				
	Urinary tract infection	17(60%)	9(60%)	0.63
	Urinary incontinence	4 (14%)	1(6%)	0.45
	Progressive hydronephrosis	6(21%)	4(24%)	0.69
	Urethral obstruction	1(3,5%)	2(12%)	0.23
Mean age at surgery (months) / SD*		15.5 / 47.7	17/39	0.43
Median follow-up (months)		84	42.5	0.003
**Unplanned surgery diagnosis**		8 (30.7%)	1 (6%)	0.07
	**DE NOVO** reflux to lower pole moiety	1 (12,5%)	1(100%)	0.64
	Ureteral stump sindrome	6 (75%)	0	0.04
	Persistent urethral obstruction (ureterocele)	1 (12.5%)	0	0.45
**Need of further surgery**				
	Endoscopic injection to remnant moitey	1(12.5%)	0	
	Uretero-vesical reconstructive surgery	2(25%)	1(100%)	
	Laparoscopic ureteral stump ressection	5(62.5%)	0	

* Standard deviation.

Overall complication was 20%. A total of 8 patients required unplanned further surgery in Group-1 (30%) whereas only 1 patient required unplanned surgery in group-2 (6%). One patient in each group presented with de novo reflux to the lower remnant pole and were treated with endoscopic injection of bulking agent and uretero-vesical reconstructive surgery (UVRS), respectively. This symptomatic patient in group-2 was further investigated and a dysfunctional voiding pattern was diagnosed, being addressed with diet improvement and frequent voiding instructions after the surgical repair. No lesions of lower moiety ureter neither lower pole loss of function was identified during or after surgery for both groups.

Although the global rate of re-do surgeries did not reach a statistically significant difference (p=0.07), subgroup analysis showed that Group-1 presented more DUSS (with at least one of previously described symptoms) requiring surgery during follow-up than group-2 (p=0.04), requiring laparoscopic ureteral stump resection in 5 patients and open UVRS in one. Another patient from group-1 persisted with urethral obstruction (due to non-collapsed ureterocele) and was submitted to successfully UVRS. Detailed data and need for further surgery are presented in Table-1. Factors possibly affecting complications incidence, such as ureterocele or ectopic ureter, did not differ between groups (p=0.72 and p=0.78).

## DISCUSSION

The management of symptomatic duplex kidney with severely impaired upper pole has been a recurrent issue in literature. Proximal approach with upper pole nephrectomy or distal (UVRS) approach, addressing the UVJ and preserving the upper moiety, are options to treat these patients (8).

Classic upper pole nephrectomy is a widely accepted procedure to correct symptomatic duplex systems with poor functioning moieties, but several complications of this approach were described in literature: loss of function in the remaining lower pole as high as 8% (11), ureteral stump complications around 16% (9) and de novo reflux to lower pole in up to 25% (5, 12). Main advantages of this approach when performed laparoscopically are those of the minimally invasive approach, with reduced pain and faster recovery (10).

UVRS, on the other hand, may have a lower incidence of complication related to the ureteral ectopia, ureteral stump and ureterocele, which are known to be possible complications of top-down approach (5). This approach, however, is related to a longer urethral catheterization time, longer hospital stay, bladder spasms, hematuria and potentially increased risk of bladder dysfunction and ureteral obstruction (4, 13, 14), although recent papers reported lower incidence of these complication (15, 16). Leaving dysplastic or hydronephrotic tissue with impaired function in situ could be harmful as well. Husmann et al. (17) reported 5% incidence of persisting urinary tract infections despite de-obstruction, and anecdotal cases of hypertension, proteinuria and renal tumors were reported.

As both ureters in duplex system kidneys run inside the same sheath deep in the pelvis, especially below iliac vessels, classic open top-down approach may limit a successful dissection to the level above iliac vessels. Development of laparoscopic knowledge and ability allows safe extended separation of the ureters, with low risk of lesion to the lower pole ureter, even when performed close to the UVJ.

Numerous authors reported the need of complementary surgery in the proximal approach between 15 and 25%, either for DUSS or new onset reflux to lower pole, especially in patients with concurrent ureterocele (5, 17), but none of these authors performed an extended distal ureteral dissection, nor cited the length of ureteral dissection to prevent such complications. In fact, most of the papers cited only partial nephrectomy as the surgical technique, which probably resulted in a long ureteral stump left behind.

Escolino et al. (10) studied the ureteral stump length after laparoscopic and retroperitoneoscopic heminephroureterectomy for duplex kidneys in symptomatic patients. Laparoscopic ureteral stump measure varied between 3 to 7mm while retroperitoneoscopic ureteral stump measured 20 to 50mm. Five years follow-up revealed no complications in the laparoscopic approach compared to two DUSS after retroperitoneoscopic surgery that required complementary surgery.

Keene et al. (8) compared proximal to distal approach and found statistically significant lower complication rate after UVRS surgery. Their proximal data were published in a previous paper, however the length of ureteral dissection was not cited (5). The complication rate after UVRS was 5% whereas in the top-down approach was 21%. In our cohort, extended distal ureteral dissection had a limited complication rate of 6%, while the limited ureteral resection group reached 30%. These results are consonant to another paper that proposed bottom-up approach as well: Gran et al. showed 8% complication rate after UVRS (4).

Our study has several limitations. Although database was maintained prospectively, a retrospective analysis was performed. The patient allocation to each group was not randomized and therefore the Group-2 had a shorter follow-up compared to Group-1. This shorter follow-up is based primarily on the surgeon's observation that a longer ureteral dissection could potentially prevent complications during upper pole nephrectomy, so there was a surgical shift towards extending the distal ureteral dissection. Another limitation of this study is that Group-2 was operated later on, with an advanced knowledge and manage on bladder bowel dysfunctions, which could eventually lead to reduced chance of UTI and stumpectomies. Further prospective randomized studies can corroborate our study hypothesis and maintain top-down approach as first-line option to treat duplex system kidneys without increasing DUSS incidence, adding minimally invasive benefits to treatment.

## CONCLUSIONS

Upper pole nephrectomy performed with extended distal ureteral dissection can prevent ureteral stump complications.
